# Dosage-sensitivity shapes how genes transcriptionally respond to allopolyploidy and homoeologous exchange in resynthesized *Brassica napus*

**DOI:** 10.1093/genetics/iyad114

**Published:** 2023-06-20

**Authors:** Kevin A Bird, J Chris Pires, Robert VanBuren, Zhiyong Xiong, Patrick P Edger

**Affiliations:** Department of Horticulture, Michigan State University, East Lansing, MI 48824, USA; Ecology, Evolution, and Behavior Program, Michigan State University, East Lansing, MI 48824, USA; Department of Soil and Crop Sciences, Colorado State University, Fort Collins, CO 80523, USA; Department of Horticulture, Michigan State University, East Lansing, MI 48824, USA; Plant Resilience Institute, Michigan State University, East Lansing, MI 48824, USA; Key Laboratory of Herbage and Endemic Crop Biotechnology, Inner Mongolia University, Hohhot, Inner Mongolia 010070, China; Department of Horticulture, Michigan State University, East Lansing, MI 48824, USA; Ecology, Evolution, and Behavior Program, Michigan State University, East Lansing, MI 48824, USA

**Keywords:** gene dosage, gene balance hypothesis, polyploidy, homoeologous exchange, genomic rearrangement, *Brassica napus*, genome evolution, dosage balance, plant genetics and genomics

## Abstract

The gene balance hypothesis proposes that selection acts on the dosage (i.e. copy number) of genes within dosage-sensitive portions of networks, pathways, and protein complexes to maintain balanced stoichiometry of interacting proteins, because perturbations to stoichiometric balance can result in reduced fitness. This selection has been called dosage balance selection. Dosage balance selection is also hypothesized to constrain expression responses to dosage changes, making dosage-sensitive genes (those encoding members of interacting proteins) experience more similar expression changes. In allopolyploids, where whole-genome duplication involves hybridization of diverged lineages, organisms often experience homoeologous exchanges that recombine, duplicate, and delete homoeologous regions of the genome and alter the expression of homoeologous gene pairs. Although the gene balance hypothesis makes predictions about the expression response to homoeologous exchanges, they have not been empirically tested. We used genomic and transcriptomic data from 6 resynthesized, isogenic *Brassica napus* lines over 10 generations to identify homoeologous exchanges, analyzed expression responses, and tested for patterns of genomic imbalance. Groups of dosage-sensitive genes had less variable expression responses to homoeologous exchanges than dosage-insensitive genes, a sign that their relative dosage is constrained. This difference was absent for homoeologous pairs whose expression was biased toward the *B. napus* A subgenome. Finally, the expression response to homoeologous exchanges was more variable than the response to whole-genome duplication, suggesting homoeologous exchanges create genomic imbalance. These findings expand our knowledge of the impact of dosage balance selection on genome evolution and potentially connect patterns in polyploid genomes over time, from homoeolog expression bias to duplicate gene retention.

## Introduction

The observation of genomic imbalance (Table 1), that changing the dosage of chromosomes or chromosome segments has a more detrimental phenotypic effect than changes to whole chromosome sets (e.g. whole-genome duplication, WGD), led to the appreciation for gene dosage changes as a powerful and important driver of gene expression abundance, quantitative trait variation, and the evolution of genomes (Birchler and Veitia 2007, 2010, 2012). These dosage changes can be divided into absolute dosage changes, related to the direct increased copy number of genes and abundance of gene product, and relative dosage changes, related to the relative copy number of genes that encode proteins connected within networks, pathways, or protein complexes and the resulting stoichiometry of their gene products (Bekaert *et al*. 2011; Conant *et al*. 2014). The finding that altered stoichiometry of gene products can have large phenotypic impacts and be highly deleterious for certain classes of genes, especially those involved in highly connected regulatory networks, and multimeric protein complexes led to the formulation of gene balance hypothesis (GBH) to explain the basis of genomic imbalance (Birchler and Newton 1981; Birchler *et al*. 2001; Makino and McLysaght 2010; Birchler and Veitia 2012). The core of the GBH proposes that changing the stoichiometry of members of networks, pathways, and protein complexes affects the kinetics, assembly, and function of the whole, which incurs negative fitness consequences (Birchler *et al*. 2005; Birchler and Veitia 2007, 2010, 2012). A valuable contribution of the GBH, and the study of relative gene dosage, is that it provides a theoretical framework to connect observations of genetic and genomic variation in the present to the past and to predictions of future evolutionary trajectories of genes and gene networks. Dosage balance selection, the selection on relative dosage to maintain the stoichiometric balance of gene products in the face of changes in gene dosage, has since been shown to influence genome evolution in important and predictable ways (Conant *et al*. 2014). This includes expectations of karyotype evolution (Xiong *et al*. 2011), duplicate gene retention and loss (Edger and Pires 2009; Makino and McLysaght 2010; De Smet *et al*. 2013; Tasdighian *et al*. 2017; Emery *et al*. 2018; Gout *et al*. 2023), gene expression (Shi *et al*. 2015; Coate *et al*. 2016; Hou *et al*. 2018; Song *et al*. 2020; Shi *et al.*  2021; Yang *et al*. 2021), and even potential mechanisms for polyploid formation (Cao *et al*. 2023).

**Table 1.** Glossary.

Term	Definition
Absolute dosage	The direct copy number of genes—absolute dosage will not necessarily track the abundance of gene product.
Allopolyploidy	Polyploidy from the hybridization of genetically diverged individuals.
Autopolyploidy	Polyploidy from the hybridization of genetically similar individuals or direct duplication of an individual's genome.
Dosage-balance selection	The selection to maintain the stoichiometric relations of the products of dosage-sensitive genes.
Dosage-sensitive genes	Genes for which a change in copy number alters expression and protein abundance and interrupts the stoichiometric balance of their gene products with those from other genes—in this study dosage-sensitivity is inferred based on patterns of duplicate gene retention.
Gene balance hypothesis	The hypothesis that the stoichiometry of members of multisubunit complexes can affect the amount of functional complete product, which in turn affects patterns of gene expression, and ultimately, the phenotype and evolutionary fitness.
Genomic imbalance	The more severe phenotypic effects of altering the number of a single chromosome or chromosome segment compared with altering the number of all chromosomes.
Homoeologs	Orthologous genes or chromosomes that diverged in the polyploid progenitors and were reunited by polyploidization.
Homoeologous exchange	Recombination between homoeologous chromosomes that swap genomic regions between subgenomes and can result in deletions, duplications, and translocations of homoeologous regions.
Homoeologous exchange response variance (HERV)	The coefficient of variation (SD/mean) of the fold change between expression in an HE region and the balanced (2:2) expression for that region (summed progenitor expression) for gene pairs affected by a HEs and within a GO term.
Homologs	Genes or chromosomes that share a common origin.
Orthologs	Genes or chromosomes from different species that arose by a speciation event.
Paralogs	Genes that arose by a duplication event.
Polyploidy	Having 2 or more complete sets of chromosomes.
Polyploid response variance (PRV)	The coefficient of variation (SD/mean) of the fold change between tetraploid expression and the midparent expression for all gene pairs from balanced (2:2) genomic regions and within a GO term.
Relative dosage	The relative copy number of genes that encode proteins connected within networks, pathways, or protein complexes—relative dosage is primarily relevant for dosage-sensitive genes.
Subgenome	A complete set of chromosomes derived from a progenitor species in an allopolyploid.

Comparative genomic studies have supported predictions from the GBH, showing that for certain classes of genes, duplicate gene retention shows biased patterns depending on whether a gene is duplicated by WGD or by small-scale duplications. This pattern of biased gene loss is best explained by dosage balance selection which selects against imbalanced stoichiometry of dosage-sensitive genes and constrains their range of possible dosage. Duplicate copies from many transcription factor families, genes involved in signaling pathways and multimeric protein complexes, and others tend to be retained more than expected after WGD, which maintains the relative dosage of these genes, and duplicates from small-scale duplications, which perturb relative dosage, tend to be retained less than expected (Blanc and Wolfe 2004; Maere *et al*. 2005; Paterson *et al*. 2006; Thomas *et al*. 2006; Freeling 2009; Edger and Pires 2009; De Smet *et al*. 2013; Conant *et al*. 2014; Li *et al*. 2016; Tasdighian *et al*. 2018). This pattern of preferential retention from WGD and loss from small-scale duplication has been called “reciprocal retention” (Freeling 2009; Tasdighian *et al*. 2018). Many of these studies have focused on ancient WGD events, where genomes have returned to a diploid-like state, leaving the immediate transcriptional impact of large-scale gene dosage changes less well understood.

Several authors have recently investigated the expression responses caused by aneuploidy and polyploidy (Coate *et al*. 2016; Hou *et al*. 2018; Song *et al*. 2020; Shi *et al*. 2021; Yang *et al*. 2021). Coate *et al*. (2016) and Song *et al*. (2020), in particular, attempt to connect observed patterns of long-term duplicate gene retention to short-term duplicate gene expression responses. They use tenets of the GBH to predict 2 patterns in short-term expression response. First, genes that are reciprocally retained after WGD (e.g. those that are highly connected in gene networks, involved in multicomponent protein complexes, etc.) should experience a change in gene expression in response to genome duplication, so that selection is able to act on dosage changes. Second, expression changes should be less variable for all genes in a network or functional class, what they call a “coordinated response.” Coate *et al*. (2016) address this question using natural soybean (*Glycine* L.) allopolyploids with an origin ∼500,000 years ago and known diploid progenitors, while Song *et al*. (2020) use 3 *Arabidopsis thaliana* autopolyploid/diploid pairs. Both studies determined that genes in reciprocally retained gene ontology (GO) terms showed a less variable expression response to polyploidy than genes from nonreciprocally retained GO terms, suggesting that genes under selection to maintain genomic balance have a constrained transcriptional response to dosage changes (Coate *et al*. 2016; Song *et al*. 2020). In the case of Song *et al*. (2020), the use of synthetic polyploids revealed that this expression response is an immediate response to altered gene dosage. Differences in expression modulation of different functional classes were also observed in synthetic polyploid and aneuploid lines of *Arabidopsis* (Hou *et al*. 2018) and maize (Shi *et al*. 2021). The use of autopolyploids or aneuploids facilitates the isolation of different types of dosage changes for investigations of genomic balance but misses the features unique to allopolyploids, which involve the hybridization of evolutionary diverged lineages and the doubling of genomic material, like homoeologous exchange (HE) or subgenome expression bias that may affect the maintenance of relative gene dosage and balanced stoichiometry.

Early studies in resynthesized allopolyploids showed extensive genetic changes in a short period of time (Song *et al*. 1995). Subsequent investigations showed major genome structural changes from the first meiosis after polyploid formation, primarily in the form of HEs in which recombination among homoeologous regions results in the partial or complete deletion and duplication of chromosomal segments (see Deb *et al*. 2023 for a recent review, see also Sharpe *et al*. 1995; Jenczeswki *et al*. 2003; Osborn *et al*. 2003; Pires *et al*. 2004; Gaeta *et al*. 2007; Nicholas *et al*. 2007, 2012; Szadkowski *et al*. 2010; Xiong *et al*. 2011; Chalhoub *et al*. 2014; He *et al*. 2017; Rousseau-Geutin *et al*. 2017; Samans *et al*. 2017; Stein *et al*. 2017; Hurgobin *et al*. 2018; Lloyd *et al*. 2018; Pelé *et al*. 2018; Mason and Wendel 2020; Bayer *et al*. 2021; Chawla *et al*. 2021; Ferreira de Carvalho *et al*. 2021; Higgins *et al*. 2021; Xiong *et al*. 2021; Orantes-Bonilla *et al*. 2022; Cao *et al*. 2023). These rearrangements continue to accumulate over time, generating genomic diversity in early polyploids (Gaeta and Pires 2010; Xiong *et al*. 2011; Mason and Wendel 2020). HEs are often destructive to the organism and meiotic stability is more frequently observed in natural polyploids compared to resynthesized and it is likely that meiotic stability is under strong selection in natural polyploid populations (Gaeta and Pires 2010; Rousseau-Gueutin *et al*. 2017; Pelé *et al*. 2018; Xiong *et al*. 2021; Gaebelein *et al*. 2019; Gonzalo *et al*. 2019; Ferreira de Carvalho *et al*. 2021). At the same time, HEs generate phenotypic novelty in resynthesized polyploids (Pires *et al*. 2004; Gaeta *et al*. 2007; Wu *et al*. 2021) and are frequently observed in natural polyploids (Chalhoub *et al*. 2014; He *et al*. 2017; Lloyd *et al*. 2018; Edger *et al*. 2019; Chawla *et al*. 2021). HEs may be under genetic control (e.g. Jenczeswki *et al*. 2003; Higgins *et al*. 2021), may affect meiotic stability (Xiong *et al*. 2021), may underlie gene presence–absence variation and agronomically valuable quantitative trait loci in *Brassica napus* (Samans *et al*. 2017; Stein *et al*. 2017; Hurgobin *et al*. 2018; Bayer *et al*. 2021), and may generate novel, chimeric transcripts as recently observed in several polyploid species including wheat, *B. napus, A. suecica*, banana, peanut, and synthetic tetraploid rice (Zhang *et al.* 2020). Less is known about the evolution of HEs.

Additionally, allopolyploid genomes must accommodate inherited and novel expression differences in homoeologous genes which often results in subgenome dominance, where expression is biased in favor of homoeologs from one progenitor genome over others (Alger and Edger 2020; Bird *et al*. 2018, 2021; Wendel *et al*. 2018). This effect is driven by the merger of evolutionarily diverged genomes, which frequently results in remodeling of epigenetic markers (Madlung *et al*. 2005; Edger *et al*. 2017; Bird *et al*. 2021), alterations in gene regulation (Chen 2007), and activation of transposable elements (Vicient and Casacuberta 2017). Importantly, there is also a continuum of polyploidy, as parental genomes within and among species can vary in evolutionary distance, and subsequent genome evolution blurs the distinction between autopolyploidy and allopolyploidy (Stebbins 1947; Leal-Bertioli *et al*. 2018; Mason and Wendel 2020; Blischak *et al*. 2023; Bomblies 2023). Subgenome expression dominance has been defined in terms of a subgenome possessing a greater amount of dominantly expressed homoeologs and has been identified in many allopolyploid species, including maize (Schnable *et al*. 2011) *Mimulus peregrinus* (Edger *et al*. 2017), garden strawberry (Edger *et al*. 2019), *B. rapa* (Cheng *et al*. 2012, 2016), and a population of resynthesized *B. napus* (Bird *et al*. 2021).

Unlike aneuploidy and polyploidy, the impact of gene expression changes from HEs or biased homoeolog expression on dosage balance is largely unexplored. There are reasons to believe HE can alter the balance of gene products in ways that entail specific evolutionary predictions from the GBH. Lloyd *et al*. (2018) found when homoeologous gene pairs have unequal expression, altering the ratio of homoeologous copies by homooeologous exchange result in dosage-dependent expression changes (i.e. proportional to the change in gene copy number). These expression changes did not accurately compensate to maintain the same level of combined homoeolog expression. Similar results have since been observed in tetraploid wheat lines (Zhang *et al*. 2022). This expression modulation from HE dosage changes resembles the protein modulation seen in aneuploid and polyploid maize lines (Birchler and Newton 1981). Therefore, in the presence of unequal homoeolog expression, dosage changes from HEs will alter expression levels of homoeologous gene pairs and, potentially, the stoichiometry of interacting gene products. Since HEs only affect a subset of the genome, the GBH predicts that this change in expression would lead to greater genomic imbalance compared to polyploidy. The GBH also predicts that the constraint on relative gene dosage from dosage balance selection would result in a more similar expression response for groups of dosage-sensitive genes affected by an HE. Whether these predicted patterns hold has not yet been explored.

We analyzed paired WGS and RNASeq data for 6 independently resynthesized and isogenic *B. napus* (CCAA) lines, which are known to accumulate large amounts of genomic rearrangement (Xiong *et al*. 2011), at 3 generations to identify HE events that resulted in altered relative dosage of genes and tested 2 predictions from the GBH regarding the transcriptional response to HEs. The first was dosage-sensitive genes and will have a less variable expression response to HE. The second was that the expression response to HEs will be more variable than the expression response to WGD. Based on previous results indicating subgenome dominance in this population of resynthesized lines favoring the *B. napus* C (BnC) subgenome (Bird *et al*. 2021), we further tested the expression response to HE and WGD to see if the transcriptional response to dosage changes differed based on which homoeolog was more highly expressed. Such results may suggest that dosage balance selection can differ between gene pairs due to the direction of subgenome-biased expression. Additionally, individuals from the first, fifth, and tenth generations were examined to see if expression responses changed over time. Our findings provide new understanding of how selection to maintain balanced stoichiometry of gene products affects gene expression and genome evolution across various modes of gene dosage changes in newly formed polyploids.

## Methods

### Sequencing data

We downloaded the whole genome sequences (WGS) and RNAseq data and files for previously identified genomic rearrangements and transcript quantification from leaf samples from Bird *et al*. (2021) at the associated Data Dryad repository https://doi.org/10.5061/dryad.h18931zjr. These previous analyses identified 26,111 syntenic ortholog pairs between the progenitor genomes, treated as homoeologous pairs from here on.

### Identification of HE events

We used the dosage assignments from Bird *et al*. (2021). Briefly, read depth ratio of WGS resequencing data for homoeologous pairs was calculated over a 50-gene sliding window with step size of 1. Homoeologous pairs were assigned 0:4, 1:3, 2:2 3:1, 4:0 based on distorted ratios of WGS reads mapping to one homoeolog over the other along a sliding window of 170 genes with a step size of 1 gene. To account for uncertainty in alignment and potential cross-mapping, the read depth was split into equal-sized quintiles to assign a dosage (0–20% as 0:4, 20–40% as 1:3, etc.). Additionally, a region was only assigned as a HE if 10 or more consecutive genes had WGS read depths within the defined quintiles.

### Expression quantification

Read count files for these samples had previously been filtered to remove lowly expressed pairs by removing gene pairs with summed TPM < 10, allowing for the potential that one copy is truly silenced. The number of homoeologous gene pairs with expression quantification in these samples ranged from 11,355 to 12,939, while the number of gene pairs affected by genomic rearrangements with expression quantification ranged from 148 for one plant in the first generation to 4,606 for a plant in the 10th generation.

### Dosage-sensitivity assignment

To leverage the well-curated gene annotations of *A. thaliana* and the close phylogenetic relationship between *A. thaliana* and the *Brassica* genus, we assigned our *Brassica* gene pairs to the GO category of their *A. thaliana* ortholog. Orthologs between *A. thaliana* and *B. oleracea* were identified with Synmap (Lyons *et al*. 2008) on CoGe (Lyons and Freeling 2008), and the *A. thaliana* GO annotations were directly assigned to the *B. oleracea* orthologs and from *B. oleracea* to the *B. rapa* syntelogs. Next, we used the GO term dosage response assignments (dosage-insensitive and dosage-sensitive) from Song *et al*.'s (2020) analysis of gene retention patterns of *A. thaliana* genes to classify our syntenic homoeologs as belonging to dosage-sensitive and dosage-insensitive GO terms. *Arabidopsis* genes, their associated GO terms and classification from Song *et al*. (2020), and the identified *B. oleracea* orthologs can be found in the Supplementary File S1.

### HE response variance

We included only syntenic homoeolog pairs that diverged from 2:2 dosage ratio (e.g. gene pairs with read-depth ratio less than 0.4 or greater than 0.6), as identified by Bird *et al*. (2021), to investigate the effects of gene dosage changes. Previous cytogenetic analysis of these lines revealed substantial aneuploidy and partial chromosomal duplication/deletion, especially among the most syntenic chromosome pairs like A1/C1, A2/C2, and C9/A10 (Xiong *et al*. 2011). To eliminate confounding effects of these kinds of rearrangements, we checked our lines for regions of skewed read depth that covered the majority or entirety of a chromosome. We fully excluded chromosomes where the majority of plants showed these large regions of skewed read depth ratios and individually removed cases where large skewed ratios were seen for chromosomes in one sample. Plots of read depth ratios along the genome for each line and generation are shown in Supplementary Figs. 1–6. This resulted in the removal of syntenic homoeologs from chromosomes A1/C1, A2/C2, and C9/A10 from all lines and chromosome C4 only for line EL-1100 at generation 10. Parental expression was taken from Bird *et al*. (2021), where RNAseq from independent libraries of *B. rapa* acc. IMB218DH and *B. oleracea* acc. TO1000DH were each aligned separately to the “in silico polyploid” concatenated reference genome comprised of the *B. rapa* acc. R500 genome (Lou et al., 2020) with SNP correction using IMB218DH resequencing data and the *B. oleracea* TO1000 reference genome (Parkin et al., 2014). This was the same concatenated reference genome used to align the RNAseq data from the resynthesized lines.

We defined the expression response to HE as the fold change of the summed homoeolog pair expression and the parental expression $\left(\frac{\mathrm{Ex}p_{BnC}+\;\mathrm{Ex}p_{BnA}}{\mathrm{Ex}p_{B.\;oleracea}+\;\mathrm{Ex}p_{B.\;rapa}\;}\right)$. Following the approach of Coate *et al*. (2016) and Song *et al*. (2020), we and calculated the coefficient of variation of this expression response $\left(\frac{\sigma_{\exp}}{\mu_{\exp}}\right)$ and termed it the HE response variance (HERV). Statistical analysis was done with a Kruskal–Wallis test applied by the function stat_compare_means() in the R package ggpubr v.0.04.0 (R Core Team 2020; Kassambara 2020). We calculated HERV only for GO terms that contained more than 20 genes. When analyzing the response to polyploidy among different homoeolog expression biases, the expression bias of progenitor orthologs was used. The classification of biased homoeologs was taken from Bird *et al*. (2021) who used a cutoff of log2-fold change of 3.5 and −3.5 to classify a homoeolog as more dominantly expressed. Previous analysis showed that for over 70% of homoeologs, all 6 resynthesized *B. napus* lines shared the same homoeolog expression bias as the parents (Bird *et al*. 2021).

### Expression response to polyploidy

When investigating the dosage response to polyploidy, we limited our analysis to the syntenic homoeologous genes identified as being in a 2:2 dosage ratio. We created our dataset by combining data across individuals, selecting gene pairs in 2:2 for a particular individual sample. We did not require that a gene pair was in 2:2 dosage in every line. We calculated expression response to polyploidy for each gene pair, defined as the fold change of polyploid expression for a 2:2 syntenic homoeolog pair and the midparent expression of the progenitor ortholog pair $\left(\frac{\mathrm{Ex}p_{B.\;oleracea}+\;\mathrm{Ex}p_{B.\;rapa}}{2}\right)$. We used the same parental expression data from Bird *et al*. (2021) as the HERV analysis. We applied the same approach as Coate *et al*. (2016) and Song *et al*. (2020) and focused on the coefficient of variation of expression response $\left(\frac{\sigma_{\exp}}{\mu_{\exp}}\right)$, which we similarly termed the polyploid response variance (PRV). The Kruskal–Wallis implementation from ggpubr (Kassambara 2020) was used again for statistical analysis. As for the previous analysis, we only included GO terms with 20 or more genes and defined homoeolog expression bias in terms of expression bias in parental orthologs.

## Results

### Homoeologous pairs of dosage-sensitive genes show a less variable expression response to HE, except when the *B. napus* A homoeolog is more highly expressed

To assess how the selection for relative dosage may affect the expression response to gene dosage changes from HE, we used the dosage-sensitivity gene class assignments for *A. thaliana* from Song *et al*. (2020). As per Song *et al*. (2020), class I GO terms are putatively dosage-insensitive and class II are putatively dosage-sensitive, and these classes are based on the observed reciprocal retention (overretention after WGD and underretention after small-scale duplication) of genes from the investigated GO terms following the At-alpha duplication event in the Brassicaceae. Similar patterns of reciprocal retention have been identified across angiosperms. While GO terms are broad and will likely include both dosage-sensitive and dosage-insensitive genes, Song *et al*. (2020) previously showed that certain GO terms result in qualitatively similar results as metabolic networks, protein-protein networks, and gene families. To leverage the superior annotation quality of *A. thaliana*, the orthologs in *B. rapa* and *B. oleracea* were assigned to the dosage-sensitivity GO classes of their *Arabidopsis* ortholog. These dosage-sensitivity assignments were used to assess how expression response differs between classes in the resynthesized allopolyploids. GO terms were then filtered, so that only those with 20 or more genes in our dataset were included in the analysis

The extensive genomic rearrangements observed in this population of resynthesized lines (Xiong *et al*. 2011; Bird *et al*. 2021) provide an opportunity to test for the first time whether gene expression changes from HE events show signs of the constraint on relative dosage from dosage balance selection that is predicted by the GBH. Using the published results from Bird *et al*. (2021), we focused on genomic regions identified as not being in 2:2 dosage, representing genomic rearrangements with 0:4, 1:3, 3:1, and 4:0 dosage ratios (BnC:BnA). To avoid the inclusion of likely aneuploidy events, genes on chromosomes that frequently showed dosage changes for the entirety or majority of the chromosome were excluded. This affected chromosome pairs 1A/1C, 2A/2C, and 10A/9C (Supplementary Figs. 1–6). With this dataset of gene pairs affected by putative HE events, we compared their expression with a balanced dosage state which we represented as the summed expression of the progenitor orthologs. It should be noted this approach did not normalize RNA with exogenous spike-in as other studies have, meaning values reported are relative gene expression levels rather than the absolute expression response. Although we cannot assign expression responses to categories like dosage-dependent or compensation as previous studies have (Hou *et al*. 2018; Song *et al*. 2020; Shi *et al*. 2021) , we can investigate the relative change in expression and test to see if it matches predictions laid out by the GBH. This type of analysis should be robust to the issues caused by the lack of an exogenous spike-in and has been previously employed in expression comparisons of natural allopolyploid and diploid species, without the use of spike-ins (Coate *et al*. 2016).

**Fig. 1.**
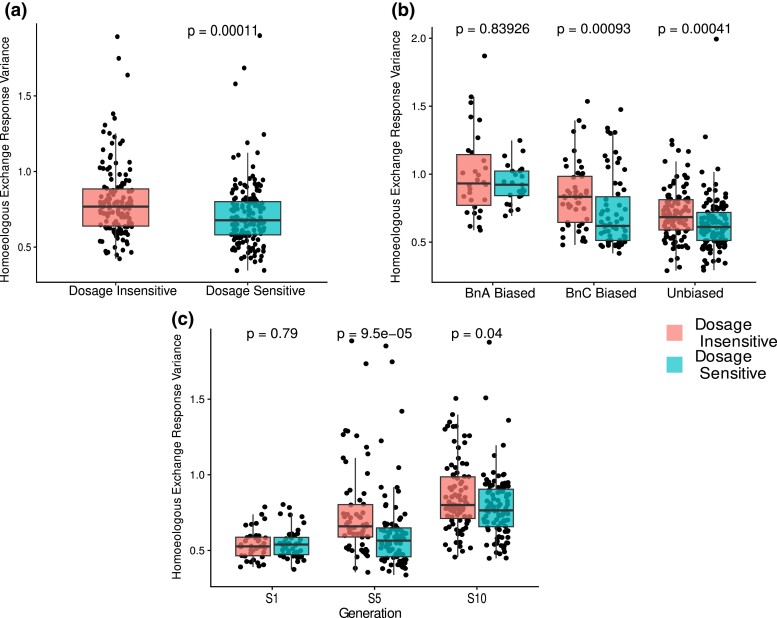
Expression changes from HE reflect predictions from the gene balance hypothesis. HERV (coefficient of variation of dosage response from HE) for all dosage imbalanced homoeologs in all 16 isogenic polyploid plants broken down by a) only putatively dosage-insensitive (class I) and dosage-sensitive (class II) GO terms from Song *et al*. (2020), b) dosage-sensitivity classes and subgenome dominance relationship in parental lines, and c) dosage-sensitivity classes and generation. *P*-values represent results of Kruskal–Wallis test of PRV between dosage-sensitive and dosage-insensitive GO terms. In all plots, individual dots represent a GO term, restricted only to GO terms that were represented by 20 or more genes in our dataset.

We investigated the extent that expression responses from HEs differ among the identified dosage-sensitive and dosage-insensitive GO terms (Fig. 1). We looked at the expression response of gene pairs in a given GO term, only for those gene pairs affected by a HE event. We used the coefficient of variation of this expression response, which we call the HERV, to assess how variable the expression response was for genes from dosage-sensitive and dosage-insensitive GO terms. After filtering GO terms with fewer than 20 genes represented in our dataset, we had 305 GO terms, with 142 dosage-insensitive and 163 dosage-sensitive. Across all lines, genes belonging to putatively dosage-sensitive GO terms showed significantly lower HERV, indicating a less variable expression response than genes from putatively dosage-insensitive GO terms (Fig. 1a; Kruskal–Wallis test, *P* = 0.00011).

It is possible that the higher variation in expression response of genes in dosage-insensitive GO terms may be may an artifact of differences in average expression of genes in dosage-sensitive and dosage-insensitive GO terms, since there is generally lower variance for more highly expressed genes (Conesa *et al*. 2016; Mortazavi *et al*. 2008).

To rule this out, we compared average transcripts per million (TPM) of homoeolog pairs for GO terms assigned as dosage-insensitive (class I) and dosage-sensitive (class II) with a Kruskal–Wallis test to make sure genes in dosage-sensitive GO terms did not have significantly higher expression compared to dosage-sensitive GO terms. For this dataset, our results showed that dosage-sensitive (class II) GO terms had significantly lower expression on average compared to dosage-insensitive (class I; *P* = 0.033; Supplementary Fig. 7). These results are similar to what Song *et al*. (2020) found for their Arabidopsis polyploids and should provide strong support that our results are not an artifact of dosage-sensitive genes being more highly expressed.

Using an allopolyploid gave us the opportunity to observe if the transcriptional response of dosage-sensitive and dosage-insensitive genes varies based on homoeolog expression bias. Such a result may suggest that the dosage constraint on homoeologous pairs from dosage balance selection can differ depending on which copy is more highly expressed. Previous transcriptomic analysis of these resynthesized lines from Bird *et al*. (2021) identified significantly biased homoeolog pairs defined as a log2-fold change greater than 3.5 or less than −3.5 and observed more homoeolog pairs with expression biased toward the BnC subgenome, which was dubbed the dominant subgenome (Bird *et al*. 2021). We compared the dosage-sensitive and dosage-insensitive GO terms, this time only including gene pairs with particular homoeolog expression bias in GO terms. This resulted in 3 datasets: expression response of GO terms only considering expression data for gene pairs with expression biased toward the BnC subgenome, pairs only with expression biased toward the *B. napus* A (BnA) subgenome, and pairs with no expression bias. Expression bias of the gene pair was based on the expression relationship of the parental orthologs. Previous analyses by Bird *et al*. (2021) found these parental expression differences to match homoeolog expression bias in all 6 lines for over 70% of homoeologous gene pairs (Bird *et al*. 2021).

When broken down by direction of homoeolog expression bias, there were 55 GO terms (30 dosage-insensitive and 25 dosage-sensitive), after filtering, for pairs biased toward the nondominant BnA subgenome, 112 GO terms (49 dosage-insensitive and 63 dosage-sensitive) for pairs biased toward the dominant BnC subgenome, and 239 (105 dosage-insensitive and 134 dosage-insensitive) for gene pairs without expression bias. We found that homoeologous gene pairs with expression biased toward the dominant BnC subgenome (Kruskal–Wallis test, *P* = 0.00093) and unbiased gene pairs (Kruskal–Wallis test, *P* = 0.00041) show significantly lower HERV in dosage-sensitive GO terms than dosage-insensitive GO terms, while pairs with expression biased toward the BnA subgenome did not show a significant difference between classes (Fig. 1b; Kruskal–Wallis test, *P* = 0.83926). Thus, we present evidence that the expression response of dosage-sensitive gene pairs differs depending on which homoeolog is more highly expressed.

**Fig. 2.**
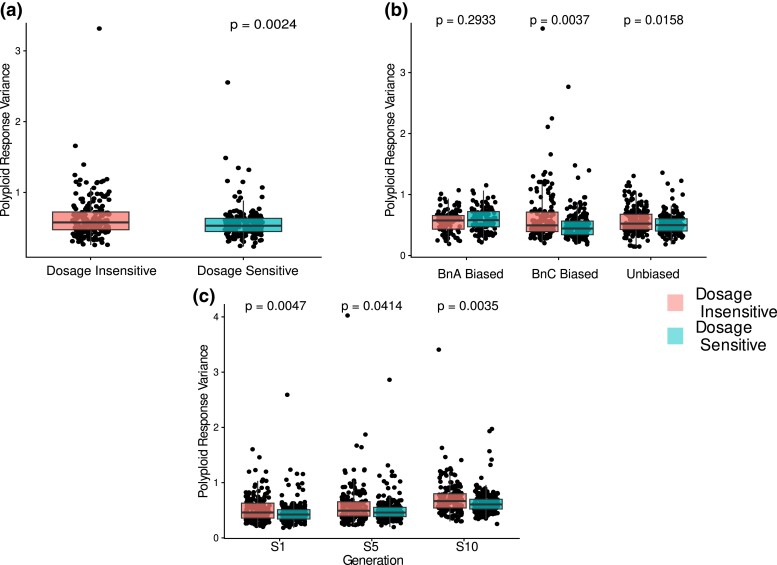
Expression changes from allopolyploidy reflect predictions from the GBH. PRV (coefficient of variation of dosage response) for all 2:2 balanced homoeologs in all 16 isogenic polyploid plants broken by a) only putatively dosage-insensitive (class I) and dosage-sensitive (class II) GO terms from Song *et al*. (2020), b) dosage-sensitivity classes and subgenome dominance relationship in parental lines, and c) dosage-sensitivity classes and generation. *P*-values represent results of Kruskal–Wallis test of PRV between dosage-sensitive and dosage-insensitive GO terms. In all plots, individual dots represent a GO term, restricted only to GO terms that were represented by 20 or more genes in our dataset.

When analyzing expression response by generation, there were 80 GO terms (36 dosage-insensitive and 44 dosage-sensitive) that passed filtering for generation 1, 148 (63 dosage-insensitive and 85 dosage-sensitive) for generation 5, and 187 (87 dosage-insensitive and 100 dosage-insensitive) for generation 10. We found that there was not a significant difference in HERV between dosage-sensitive and dosage-insensitive GO terms at the first generation (Fig. 1c, Kruskal–Wallis test, *P* = 0.79), but dosage-sensitive and dosage-insensitive GO terms did show different HERV at the fifth and tenth generations (Fig. 2c, Kruskal–Wallis test, *P* = 9.5 × 10^−5^, *P* = 0.04). We also found that HERV increased over time with dosage-sensitive and dosage-insensitive GO terms showing mean HERV of 0.661 and 0.502, respectively, in generation 1 and increasing to 0.828 and 0.697, respectively, in generation 10.

### Homoeologous pairs of dosage-sensitive genes show less variable expression response to WGD, except when the BnA homoeolog is more highly expressed

We further investigated the transcriptional response to dosage changes and variation by subgenome expression bias by analyzing the relative gene expression change for individual homoeologous gene pairs in 2:2 dosage. We took the fold change of the summed transcript count for homoeologous gene pairs in the allopolyploid individuals and midparent value of the progenitor orthologs. We used the PRV measure from Song *et al*. (2020) and Coate *et al*. (2016), defined as the coefficient of variation of the relative expression response, to assess how variable the expression response to polyploidy is in the different gene groups. These analyses allowed us to establish the expression response to polyploidy in a newly formed allopolyploid and further explore and validate the findings about homoeolog expression bias in the HERV analysis.

Analyzing data across all lines and filtering out GO terms with fewer than 20 genes, we had a final count of 376 GO terms of which 181 were classified dosage-insensitive and 195 were dosage-sensitive. As observed previously in resynthesized autopolyploids and natural *Glycine* allopolyploids, the PRV was significantly lower (i.e. the expression response was less variable) in genes from GO terms in the dosage-sensitive class compared to the dosage-insensitive class (Kruskal–Wallis test, *P* = 0.0024; Fig. 2a). We again checked for expression differences between dosage-sensitive and dosage-insensitive groups of genes. For this dataset of homoeologous pairs at 2:2 dosage, our results showed that dosage-sensitive (class II) GO terms had significantly lower expression on average compared to dosage-insensitive (class I; *P* = 0.0085; Supplementary Fig. 8). This again supports that our results are not due to differences in expression between genes in the class I and class II GO terms.

We next sought to replicate the above results showing a less variable expression response for dosage-sensitive gene pairs when the BnA homoeolog was more highly expressed, this time using PRV. After filtering out GO terms with fewer than 20 genes, there were 274 GO terms (113 dosage-insensitive, 124 dosage-sensitive) for gene pairs biased toward the nondominant BnA subgenome, 330 GO terms (156 dosage-insensitive and 174 dosage-sensitive) for pairs biased toward the dominant BnC subgenome, and 374 GO terms (179 dosage-insensitive and 195 dosage-sensitive) for genes not biased toward either subgenome. We again found that pairs with expression biased toward the BnC, or with unbiased expression, showed a significant difference between PRV of dosage-sensitive and dosage-insensitive GO terms as above (Kruskal–Wallis test, *P* = 0.0037; *P* = 0.0158, respectively). As before, gene pairs biased toward the BnA subgenome showed no significant difference in PRV between dosage-sensitive and dosage-insensitive GO classes (Kruskal–Wallis test, *P* = 0.2933; Fig. 2b). These results provide further support that the expression response of dosage-sensitive genes differs depending on which homoeolog is more highly expressed.

When broken down by generation, there were 375 GO terms (180 dosage-insensitive and 195 dosage-sensitive) that passed filtering for generation 1, 368 GO terms (174 dosage-insensitive and 194 dosage-sensitive) for generation 5, and 362 GO terms (172 dosage-insensitive and 190 dosage-sensitive) for generation 10. In all 3 generations, dosage-sensitive GO terms have significantly lower PRV than dosage-insensitive GO terms (Fig. 2c; Gen 1 *P* = 0.0047, Gen 5 *P* = 0.0414, Gen 10 *P* = 0.0035). We observed an increase in the coefficient of variation over time, with both dosage-sensitive and dosage-insensitive showing higher PRV in generation 10 than in the first generation (Fig. 2c). Notably, in generation 10, the dosage-sensitive GO terms show higher mean polyploidy response variance than dosage-insensitive GO terms in the first generation.

### Expression response to HE is more variable than the expression response to WGD

**Fig. 3.**
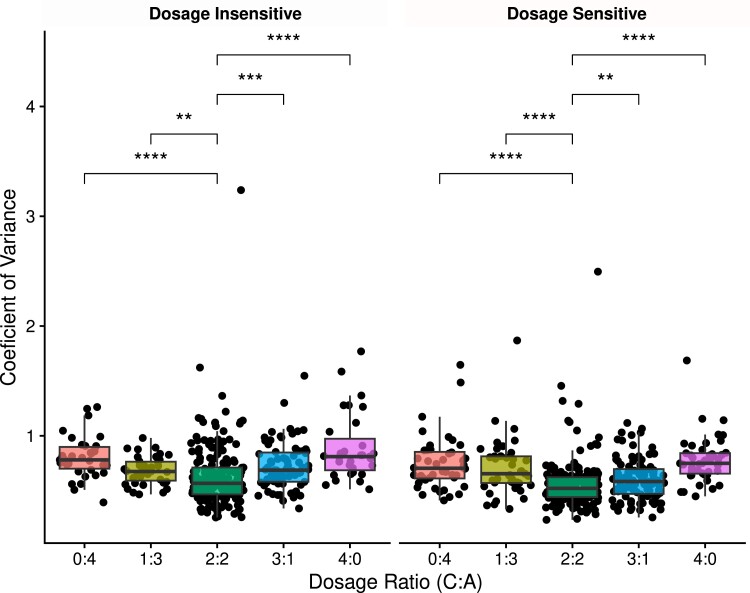
Expression responses to HE are more variable than the response to polyploidy. Comparison of the coefficient of variation for the expression response to HEs (dosages 0:4, 1:3, 3:1, and 4:0) and allopolyploidy (dosage 2:2) for dosage-insensitive GO terms (left) and dosage-sensitive GO terms (right) combined across all lines and generations. Individual dots represent a GO term, restricted only to GO terms that were represented by 20 or more genes in our dataset. Asterisks represent *P*-value of pairwise Wilcoxon tests between an HE dosage group (0:4, 1:3, 3:1, 4:0) and the 2:2 group, representing the polyploidy expression response. Asterisks represent significance levels as follows: *P ≤ 0.05, **P ≤ 0.01, ***P ≤ 0.001, and ****P ≤ 0.0001.

The GBH predicts that dosage changes that alter only a subset of genes in a pathway or protein complex will produce genomic imbalance that can reduce organismal fitness, while changes that alter the dosage of the entire genome will maintain the necessary stoichiometric balance of gene products. Given that HEs result in dosage and expression changes for only some regions of the genome, they are predicted to produce genomic imbalance. While we do not have phenotypic data to directly investigate genomic imbalance, previous work has shown a greater variation in expression response to aneuploidy than polyploidy in *A. thaliana* (Hou *et al*. 2018). These authors argued the patterns of expression modulation exhibited by aneuploids reflected the same principles of genomic balance as is observed at the phenotypic level. Therefore, investigating patterns of expression between genes affected by HE and polyploidy may still shed light on whether HEs produce genomic imbalance. To test for signs of genomic imbalance, we compared the coefficient of variation for the expression response to the polyploidy and HE datasets to see if the HE expression response was more variable (Fig. 3).

First, we compared the proportion of gene pairs belonging to dosage-sensitive and dosage-insensitive GO terms in all 16 individuals for the polyploidy and HE analysis. For the polyploid analysis, the mean proportion of genes belonging to dosage-insensitive GO terms is 0.554, while it is 0.544 for the HE analysis. This difference in the proportion of class I and class II GO terms between the PRV and HERV analysis was not statistically significant (χ^2^ test, *P* = 0.983). Even considering the direction of difference in the proportion of dosage-insensitive genes, a greater proportion of gene pairs having dosage-insensitive GO terms would be predicted to result in a higher coefficient of variation. Instead, we found a significantly higher coefficient of variation from all pairwise comparisons between the expression response to HEs of different directions and dosage and the expression response to polyploidy. This was the case for both dosage-sensitive and dosage-insensitive GO terms (Fig. 3; Wilcoxon test, *P* < 0.01). Additionally, the results appear to show an additive pattern where the coefficient of variation in expression response becomes larger and more significantly different from the coefficient of variation of the polyploidy expression response for the 0:4 and 4:0 HEs, which represent larger dosage changes than 1:3 and 3:1 HEs. These results further support the prediction that HEs can create genomic imbalance, because a more variable expression response is predicted to translate to more variation in the final amount of interacting gene products, which disrupts the stoichiometry of the system.

Finally, the significantly different expression response to HEs and polyploidy-induced dosage changes in all comparisons are strong evidences that the patterns observed for HE-induced dosage changes are, at least partially, distinct from the effects of polyploidy-induced dosage change, and our results from previous sections are not merely an artifact of our HE analysis picking up the effects of dosage changes caused by allopolyploidy or trans-dosage effects of aneuploidy.

## Discussion

### Genomic balance and the evolution of HEs

HEs have long been recognized as an engine of phenotypic diversity and novelty in newly formed polyploids (Pires *et al*. 2004; Gaeta *et al*. 2007; Xiong *et al*. 2011; Rousseau-Guetin *et al*. 2017; Stein *et al*. 2017; Leal-Bertioli *et al*. 2018; Lloyd *et al*. 2018; Mason and Wendel 2020; Ferreira de Carvalho *et al.* 2021; Wu *et al*. 2021; Bomblies 2023). Our analysis of genomic rearrangements and HEs in resynthesized *B. napus* confirmed at higher resolution the extensive rearrangements in these lines (Gaeta *et al*. 2007; Xiong *et al*. 2011). Investigations of genomic balance and dosage-sensitivity have predominantly focused on polyploidy and aneuploidy as the sources of gene dosage alteration (Hou *et al*. 2018; Shi *et al*. 2021; Yang *et al*. 2021). However, HEs, which alter the dosage ratio of parental chromosome segments, have also been shown to produce dosage-dependent expression changes that perturb the total expression of a gene pair (Lloyd *et al*. 2018). We tested 2 predictions from the GBH regarding the transcriptional response to HEs. The first is that the dosage balance selection to maintain the stoichiometry of gene products will result in dosage-sensitive genes having a less variable expression response to HE. The second is that, due to the perturbation to stoichiometry from HEs, the expression response to HEs will be more variable than the expression response to WGD.

We found that expression response to HEs matches those previously observed in response to WGD (Fig. 1a; Coate *et al*. 2016; Song *et al*. 2020). Gene expression responses from dosage-sensitive GO terms are less variable than those from dosage-insensitive GO terms, as predicted by the GBH. However, we also saw the difference in expression response between dosage-sensitive and dosage-insensitive genes was not present for homoeolog pairs with expression biased toward the nondominant BnA subgenome. When comparing expression response variation from HEs to polyploidy, we observe significantly higher variation in the expression response to HEs (Fig. 3). Similar results from comparisons of expression modulation from aneuploidy and polyploidy have been taken as evidence for genomic imbalance and an ultimate explanation for the cause of the greater phenotypic impacts and fitness cost of aneuploidy (Hou *et al*. 2018; Shi *et al*. 2021). These results similarly support a genomic imbalance arising from HEs and raise the possibility that a similar fitness cost due to genomic imbalance exists for HEs, though the magnitude relative to other fitness costs like meiotic instability cannot be determined here. Such results have not been reported before, to our knowledge, and provide strong evidence that expression response of dosage-sensitive genes to HEs is affected by dosage balance selection.

If HEs evolve in ways predicted by the GBH, then we might expect dosage balance selection to disfavor HEs containing dosage-sensitive genes, producing biases in the gene functions surviving HEs that are similar to those for small-scale duplications. Indeed, Hurgobin *et al*. (2018) and Bayer *et al*. (2021) identified a significant degree of gene presence–absence variation in *B. napus* arising from HEs. Presence–absence variation was negatively associated with membership in protein–protein interaction networks (Bayer *et al*. 2021) and positively associated with GO terms related to plant defense and stress pathways (Hurgobin *et al*. 2018). They also observed several HEs generating presence–absence variation in paralogs of the large gene family *FLC* (Flowering Locus C), which regulates flowering time. Analysis of expression dynamics of *FLC* paralogs in *B. napus* showed that while *FLC* paralogs are dosage-sensitive, the selection on dosage balance acts on overall *FLC* gene family expression allowing compensatory drift (Thompson *et al*. 2016) and expression divergence (Calderwood *et al*. 2021). This *FLC* example shows that the interplay of HE and dosage balance may be highly dynamic depending on the gene family in question. The effect of dosage-sensitivity on expression response to HEs observed here may also help understand the mechanisms reported by Lloyd *et al*. (2018), which observed a tendency of transcriptional compensation of older HE events in natural *B. napus*.

Finally, selection for dosage balance may also drive subgenome biases in the direction of HE. For example, Edger *et al*. (2019) proposed that the selection for stoichiometric balance of gene products could explain the overwhelming bias in direction of HE, favoring the dominant subgenome, in the octoploid strawberry genome. These results might help explain why natural *B. napus* lines tend to show bias in favor of HE events decreasing copies of C subgenome regions (Nicholas *et al*. 2012; Chalhoub *et al*. 2014), as perturbing the level of gene products among only a fraction of the members of interacting units is expected to incur a fitness cost similar to aneuploidy. Our results show that the effect of dosage-sensitivity on the transcriptional response to dosage changes differs for gene pairs depending on which homoeolog is more highly expressed. If the less variable expression response of dosage-sensitive genes is truly a result of the dosage balance selection on homoeologous pairs to maintain proper stoichiometry of their gene products, this raises the possibility that dosage balance selection acts on homoeologous pairs differently depending on which copy is more highly expressed. This pattern suggests that the changes to homoeologs from one subgenome, in this case the dominant BnC subgenome, are more likely to contribute to stoichiometric imbalance.

It is worth noting, we were not able to completely rule out expression changes from other gene loss or silencing processes, like partial chromosomal deletion or duplication or DNA methylation. However, it is unlikely that our results are driven primarily by these other factors. Gaeta *et al*. (2007) analyzed genomic, epigenomic, and transcriptomic changes in this population in the fifth generation using gel-based markers, where 71% of genetic RFLP (restriction fragment length polymorphism) marker deletions were accompanied by intensifications by homoeologous markers from the other subgenome, supporting HE events in the majority of cases. Gaeta *et al*. (2007) also reported no correlation between epigenomic markers and expression changes, measured by cDNA-AFLP (amplified fragment length polymorphism) markers, in the siblings of this population. Similarly, Hou *et al*. (2018) looked at gene expression and DNA methylation changes in an aneuploid series of Arabidopsis and did not identify consistent changes in DNA methylation in any context from aneuploidy. They interpreted this as evidence that genomic imbalance and its expression effects were not mechanistically caused by methylation changes. Still, future work combining cytogenetics, genomics, and epigenomics more directly will provide a clearer picture of the kinds of expression patterns reported here and other factors like non-HE genomic rearrangement and epigenetic changes.

### The effect of dosage-sensitivity on expression responses differs depending on which subgenome is more highly expressed

In both of our HERV and PRV analyses, we observed evidence of the difference in expression response between dosage-sensitive and dosage-insensitive genes being absent for homoeologous pairs with expression biased toward the nondominant subgenome. This may indicate that the constraint from dosage balance selection differs between dosage-sensitive gene pairs depending on the direction of their homoeolog expression bias. Previous analysis of these resynthesized lines showed that homoeologous pairs biased toward the BnC subgenome, which was the maternal contributor and called the dominant subgenome, were more connected in a protein–protein interaction network, while pairs with expression biased toward the paternal BnA subgenome showed no such enrichment for connectivity (Bird *et al*. 2021). This lack of connectivity may explain why putatively dosage-sensitive genes with biased expression toward the nondominant subgenome do not show less variable expression; without high connectivity in gene networks, they do not experience strong dosage-balance selection. Bird *et al*. (2021) speculated that this enrichment was driven by interactions between the nuclear and organellar genomes, given the functional enrichment for mitochondria, chloroplast, and cytoplasm among the protein-protein interaction (PPI) network; however, some recent work casts doubt on the impact of cytonuclear incompatibilities on this kind of response to allopolyploidy (Ferreira de Carvalho *et al*. 2019; Sharbrough *et al*. 2022). Assessing the generality of these subgenome differences in network connectivity and their relation to cytonuclear interaction will be a promising avenue for future research in this area.

It is noteworthy that we see no difference in the expression response of dosage-sensitive genes and dosage-insensitive genes for both HEs and WGD. This suggests it is a general aspect of how homoeolog expression bias and dosage-sensitive affect the expression response to dosage changes. Given the hypothesized importance of coordinated expression responses for maintaining the stoichiometry of gene products after dosage changes (Coate *et al*. 2016; Song *et al*. 2020), this may have implications for differences in long-term duplicate gene retention patterns between subgenomes. For example, over the long term, subgenome differences in expression responses might be predicted to preserve more dosage-sensitive genes from the dominant subgenome than the nondominant since dosage-sensitive gene pairs with expression biased toward the dominant subgenome will be predicted to be retained more. In line with this, Schnable *et al*. (2012) observed that biased retention of dosage-sensitive genes broke down over time, with only 50% of genes retained from one genome duplication event being retained in duplicate after a subsequent duplication event. They further observed that the lower expressed copy was more likely to be lost and proposed the lower expressed copies contribute less to final interacting gene products and so experience less purifying selection and weaker dosage constraint (Schnable *et al*. 2012). Similarly, when subgenome dominance was first described in *Arabidopsis*, the dominant subgenome was also associated with clusters of dosage-sensitive genes across the genome (Thomas *et al*. 2006).

### Implications for long-term duplicate gene evolution and the interplay of biased fractionation and reciprocal retention of duplicate genes

We propose a unified model for short-term and long-term interactions of subgenome dominance and dosage balance. This model involves: (1) greater retention of dosage-sensitive gene pairs that are biased toward the dominant subgenome due to stronger dosage balance selection and (2) the eventual divergence of duplicates over long evolutionary time and loss of nondominant homoeologs due to biased fractionation. Following duplication, gene pairs biased toward the dominant subgenome in these synthetic *B. napus* show higher connectivity in protein–protein interaction networks and functional enrichment. Dosage-sensitivity is a spectrum, most strongly correlated with the connectivity of gene products in a network or macromolecular complex (Birchler and Veitia 2012). Therefore, the less variable expression response of unbiased and dominant subgenome-biased gene pairs may be reflective of greater dosage sensitivity than pairs biased toward the nondominant subgenome. Greater dosage sensitivity predicts that these gene pairs will be retained for a longer time due to dosage balance selection to maintain proper stoichiometry, given that their loss is more likely to perturb the relative balance of interacting gene products (Freeling *et al*. 2012).

Dosage constraints on gene duplicates are not permanent and can change or subside over evolutionary time (Bekaert *et al*. 2011; Schnable *et al*. 2012; Conant *et al*. 2014). Additionally, the stoichiometry of interacting proteins is what is truly under dosage balance selection. The expression of individual paralogs can diverge so long as that stoichiometry is largely left intact, a phenomenon called compensatory drift (Thompson *et al*. 2016). In the case of subgenome dominance, one copy is contributing a greater fraction of the overall amount of that gene product. As dosage constraint weakens, deleting the more highly expressed copy will cause a greater disturbance to the stoichiometric balance (Freeling *et al*. 2012). As an extreme example, if one copy contributes 90% to total expression and the other 10%, a greater stoichiometric imbalance would be observed with interactors when losing the dominant (90%) copy. As such, the dominant copy will be under stronger purifying selection. Under compensatory drift, it is easier for the dominant copy to change expression enough to account for all or most of the gene product of a pair, thus reducing purifying selection on the nondominant copy which now contributes little-to-none to the stoichiometry balance.

This difference in purifying selection reduces the likelihood that the dominant copy is fractionated by the short-deletion mechanism postulated to drive genome fractionation in plant genomes (Woodhouse *et al*. 2010). Ultimately, genes on the nondominant subgenome will be preferentially lost, and the dominant subgenome will maintain higher gene content and more enrichment for dosage-sensitive genes—even through successive polyploid events (Woodhouse *et al*. 2014).

### Future directions

Several findings may warrant follow-up or more targeted investigation. Our comparison of HE and polyploidy response variance showed that overall gene expression was more variable in response to HE compared to polyploidy. The concept that selection for beneficial HE's may be limited by a polyploid ratchet (Gaeta and Pires 2010) or other constraints related to the negative effect of HEs on meiotic stability has been discussed in the context of allopolyploid systems such (e.g. Leal-Bertioli *et al*. 2018; Lloyd *et al*. 2018; Pelé *et al*. 2018; Edger *et al*. 2019; Ferreira de Carvalho *et al*. 2021; Higgins *et al*. 2021; Wu *et al*. 2021; Deb *et al*. 2023). Our results expand on these findings by suggesting HEs may also be selected against due to the genomic imbalance they produce. Replicating these results beyond gene classes based on GO terms, such as more fine-resolution data like metabolic pathway membership, protein–protein interaction networks, or gene families, may provide more precise estimates of effects, as functional classification based on GO terms is known to introduce heterogeneity. Additionally, this study only looks at expression in leaf tissue. Expression differences and differences in homoeolog expression bias are likely to exist across tissues. Investigation in more tissue types and looking for variation by tissue is a promising avenue of research. Future work would also benefit from approaches using spike-ins and those which can isolate HE from other trans-effects on expression, both from hybridization and aneuploidy, that could not be controlled for when assessing expression changes in this study. Such work would provide more precise estimates of the magnitude of the effect of dosage-sensitivity on expression responses to dosage changes. This improved experimental design would also help make sense of the finding that PRV and HERV for both dosage-sensitive and dosage-insensitive GO terms increase over time (Figs. 1c, 2c). Currently, it is not possible to distinguish whether this result is from changes in the strength of dosage constraints or an accumulation of interindividual variation from trans-dosage effects in the genomic background. Disentangling these 2 explanations will reveal novel insights into the dynamics of expression changes and genomic balance over short evolution time scales. One particularly interesting possibility would be exploring ways to generate or introduce HEs of a specific dosage in a controlled genetic background, allowing a more precise investigation of the effect of dosage changes and the transcriptional response.

## Conclusion

This study provides new evidence on the potential for genomic imbalance from HEs and insight into how dosage balance selection affects the gene expression changes from genomic rearrangements. These findings may help fuel more integrative genetic and evolution investigations of HE, subgenome expression dominance, and duplicate gene evolution that can leverage the vast new output of genomes with ancestral and recent polyploidy and explicit evolutionary models of ancestral subgenomes (Emery *et al*. 2018; Hao *et al*. 2021, 2022; Parey *et al*. 2022). This new avenue of investigation may help further examine evolution and epistasis as well as selection and divergence among paralogs (Qi *et al*. 2021; Conover and Wendel 2022; Kwon *et al*. 2022) and spur further integration of methods and data across phylogenomics, comparative and population genomics, and network biology (Renny-Byfield *et al*. 2017; Blischak *et al*. 2018). Such work can enhance plant breeding efforts by providing a strengthened evolutionary understanding of the consequences of gene duplicates, structural variation, relative gene dosage, and subgenome dominance (Rodríguez-Leal *et al*. 2017; Bird *et al*. 2018; Salman-Minkov *et al*. 2016; Turner-Hissong *et al*. 2020; Bayer *et al*. 2021; Bomblies 2023).

## Supplementary Material

iyad114_Supplementary_Data

## Data Availability

Raw data from this project are available on the NCBI Sequence Read Archive (SRA) Project PRJNA577908. Intermediate files can be found at https://doi.org/10.5061/dryad.h18931zjr, and code to recreate main figures and results can be found at https://github.com/KevinABird/Bird_GenomeInFlux_BNapus. Supplemental material available at GENETICS online.
