# Supplementary material for: Dosage-sensitivity shapes how genes transcriptionally respond to allopolyploidy and homoeologous exchange in resynthesized Brassica napus
Source: Genetics. 2023 Jun 20;225(1):iyad114. doi: 10.1093/genetics/iyad114 (PMC10471226; doi:10.1093/genetics/iyad114)
Supplement: iyad114_Supplementary_Data [file iyad114_supplementary_data.zip › Figure_S1_GENETICS-2023-306243.pdf]

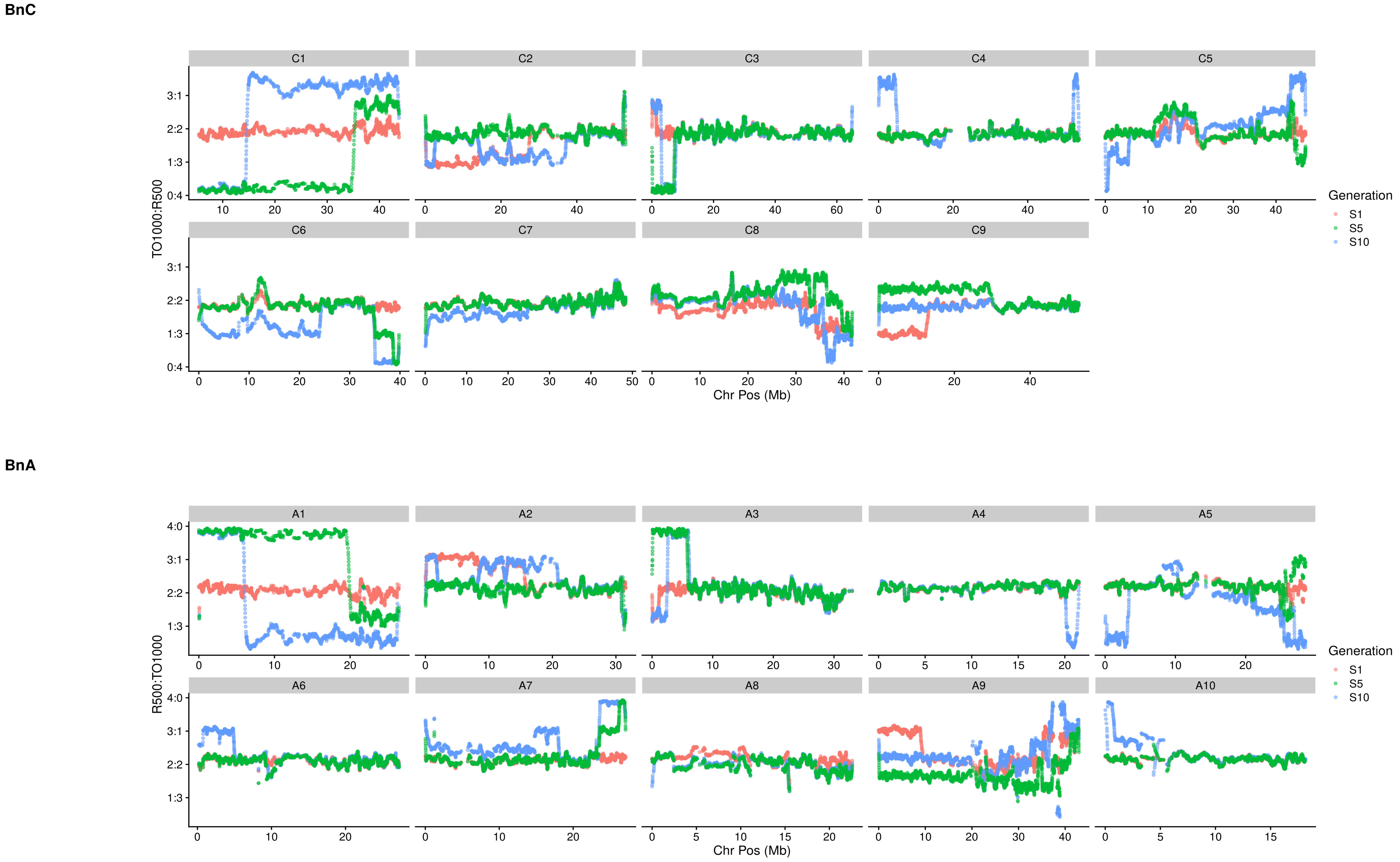

Fig S1. Mean read depth ratio of WGS reads for the BnC subgenome (top) and BnA subgenome (bottom) for individual EL100. Values represent the rolling average of the read depth ratio over a 50 gene window. Regions such as chromosomes A1/C1 where long stretches of skewed read depth ratios were taken as evidence of larger genomic changes like aneuploidy or partial duplication and excluded from subsequent analyses
