# Supplementary material for: Dosage-sensitivity shapes how genes transcriptionally respond to allopolyploidy and homoeologous exchange in resynthesized Brassica napus
Source: Genetics. 2023 Jun 20;225(1):iyad114. doi: 10.1093/genetics/iyad114 (PMC10471226; doi:10.1093/genetics/iyad114)
Supplement: iyad114_Supplementary_Data [file iyad114_supplementary_data.zip › Figure_S8_GENETICS-2023-306243.pdf]

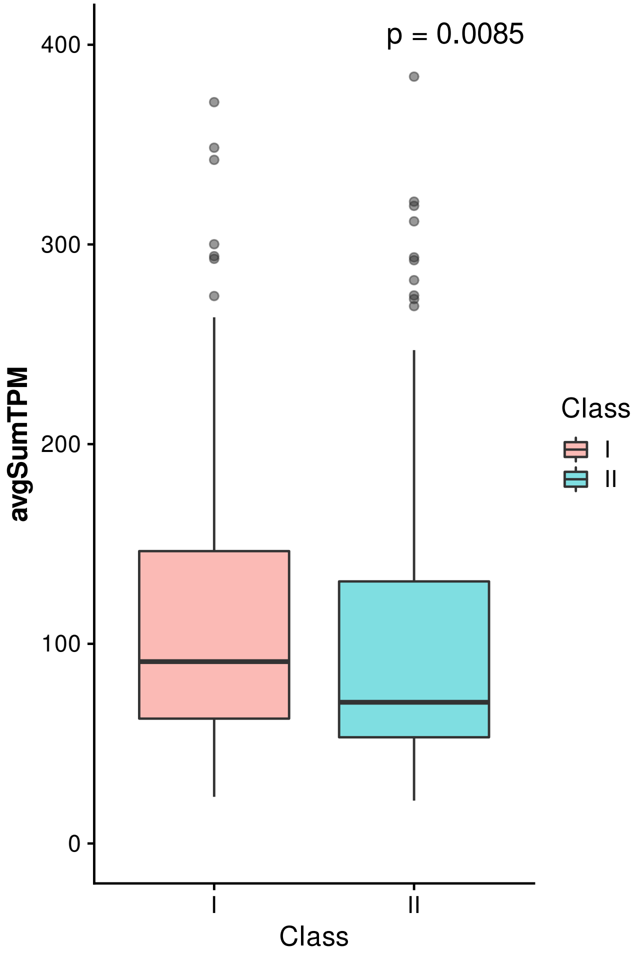

Fig S8. Comparison of average TPM of genes from Class I and II GO terms for homoeologous pairs that are at a 2:2 dosage ratio. P-value represents the results of a Kruskal-Wallis test
